# Effects of Inflammation on the Immune Microenvironment in Gastric Cancer

**DOI:** 10.3389/fonc.2021.690298

**Published:** 2021-07-23

**Authors:** Weidan Zhao, Mingqing Liu, Mingyue Zhang, Yachen Wang, Yingli Zhang, Shiji Wang, Nan Zhang

**Affiliations:** ^1^Department of Gastroenterology, The First Hospital of Jilin University, Changchun, China; ^2^Department of Intensive Care Unit, The First Hospital of Jilin University, Changchun, China

**Keywords:** inflammation, tumor microenvironment, gastric cancer, immune cell infiltration, risk score

## Abstract

**Background:**

Chronic inflammation and immune cell dysfunction in the tumor microenvironment are key factors in the development and progression of gastric tumors. However, inflammation-related genes associated with gastric cancer prognosis and their relationship with the expression of immune genes are not fully understood.

**Method:**

In this study, we established an inflammatory response model score called “Riskscore”, based on differentially expressed genes in gastric cancer. We used Survival and Survminer packages in R to analyze patient survival and prognosis in risk groups. The survival curve was plotted using the Kaplan–Meier method, and the log-rank test was used to assess statistical significance, and we performed the ROC analysis using the R language package to analyze the 1-, 3-, and 5-year survival of patients in the GEO and TCGA databases. Single-factor and multi-factor prognostic analyses were carried out for age, sex, T, N, M, and risk score. Pathway enrichment analysis indicated immune factor-related pathway enrichment in both patient groups. Next, we screened for important genes that are involved in immune cell regulation. Finally, we created a correlation curve to explore the correlation between Riskscore and the expression of these genes.

**Results:**

The prognosis was significantly different between high- and low-risk groups, and the survival rate and survival time of the high-risk group were lower than those of the low-risk group. we found that the pathways related to apoptosis, hypoxia, and immunity were most enriched in the risk groups. we found two common tumor-infiltrating immune cell types (i.e., follicular helper T cells and resting dendritic cells) between the two risk groups and identified 10 genes that regulate these cells. Additionally, we found that these 10 genes are positively associated with the two risk groups.

**Conclusion:**

Finally, a risk model of the inflammatory response in gastric cancer was established, and the inflammation-related genes used to construct the model were found to be directly related to immune infiltration. This model can improve the gastric cancer prognosis prediction. Our findings contribute to the development of immunotherapy for the treatment of gastric cancer patients.

## Introduction

In 2017, 1.2 million patients were diagnosed with gastric cancer (GC) and there were 865,000 reported deaths worldwide. Between 2007 and 2017, the incidence of gastric cancer increased by 25% (1). In China, gastric cancer is of particular concern as it was the second leading cause of cancer-related deaths in 2015 (2). Although there are many treatments available for gastric cancer, the five-year survival rate for gastric cancer is low. Therefore, it is of urgency to establish a prognostic model that can improve the gastric cancer prognosis prediction.

Since the “seed and soil” theory of tumors was published (3), our understanding of the tumor microenvironment has expanded. All of the cells that reside in the tumor microenvironment take part in the formation and development of tumors (4, 5). Studies have shown that chronic inflammation contributes to cancer progression (6). Many tumors develop following chronic inflammation or exhibit chronic inflammation throughout progression (7, 8).

The cause of gastric cancer differs among patients owing to differences in genetic and environmental factors, tumor location, histological manifestations, and other molecular features (9). However, chronic *Helicobacter pylori* infection is the main factor in the development of sporadic stomach cancer, and colonization of gastric epithelium can lead to inflammatory precancerous cascades, including chronic gastritis, atrophic gastritis, and intestinal metaplasia (10). Inflammation creates a microenvironment conducive to cellular transformation and the spread of invasive diseases. In accumulated inflammation, interactions between tumor cells and immune components perpetuate the transformed environment, inducing the transformed tumor cells to acquire mutations and epigenetic changes necessary for cell autonomy (11).

In this study, we explored the relationship between inflammation and immunity in gastric cancer to establish an inflammatory risk scoring model. Our findings serve as a basis for further studies focusing on the relationship between inflammation and the immune microenvironment of gastric cancer at the genetic level.

## Materials And Methods

### Data Collection

The clinical data and the RNA-seq data in this study were obtained from the GSEA website, GEO database and TCGA database. Immunohistochemical images from the Human Protein Atlas (HPA) (https://www.proteinatlas.org) were used to identify the protein expression levels of the corresponding genes.

### Establishment of Inflammatory Model

We identified genes that are independently associated with gastric cancer prognosis. The Riskscore of each patient was calculated as the sum of the expression of each gene in the two databases, multiplied by the expression coefficient. Taking the median of the Riskscore as the grouping standard, patients were divided into two groups (high and low) according to the risk value.

### Survival Analysis

We used Survival and Survminer packages (The R Foundation for Statistical Computing, Vienna, Austria) in R to analyze patient survival and prognosis. In total, 371 and 433 samples from the two databases were included, respectively. The patient follow-up time in the GEO database was 13 years, whereas that in TGGA database was 10 years. The survival curve was plotted using the Kaplan–Meier method, and the log-rank test was used to assess statistical significance. *P <*0.05 was a statistically significant standard.

### ROC Curve Analysis

We performed the ROC analysis using the R language package (survival, surgeon, and time ROC), to analyze the 1-, 3-, and 5-year survival of patients in the GEO and TCGA databases. The area under the ROC curve was calculated. An area under the curve of >0.5, indicates that the model can accurately predict patient survival. Patient survival in both groups is presented as risk column and a risk curve.

### Heatmap Construction

Using the PheatMap software package in R, we created a heat map showing gene expression.

### PPI Network Construction

A network of inflammatory genes was constructed using the STRING database. Next, The PPI network was visualized using Cytoscape 3.8.2, and the Cytoscape plug-in, cytoHubba, arranges the network by the number of connections, and selects the first 201 genes with the largest number of adjacent nodes for subsequent analysis.

### Cox Regression Analysis

Using the survival package in R, we performed univariate Cox regression analysis using genes associated with inflammation that were closely related to prognosis. Single-factor and multi-factor prognostic analyses were carried out for age, sex, T, N, M, and risk score.

### Correlation Between Gene and Inflammation

We screened for important genes that are involved in immune cell regulation. The ggExtra, GGPUBR, and ggplot2 packages in R were used to analyze the correlation between genes and inflammation and the expression difference between inflammation risk groups.

### GSEA

By downloading the gene symbols and HALLMARK gene set on the GSEA website, we extracted genes related to inflammation. The entire transcriptome of all tumor samples was used in the GSEA, and genomes with FDR *q* value <0.06 and nominal *P* value < 0.05 were considered statistically significant.

## Results

### Extraction of Inflammation-Related Genes

**Figure 1:**
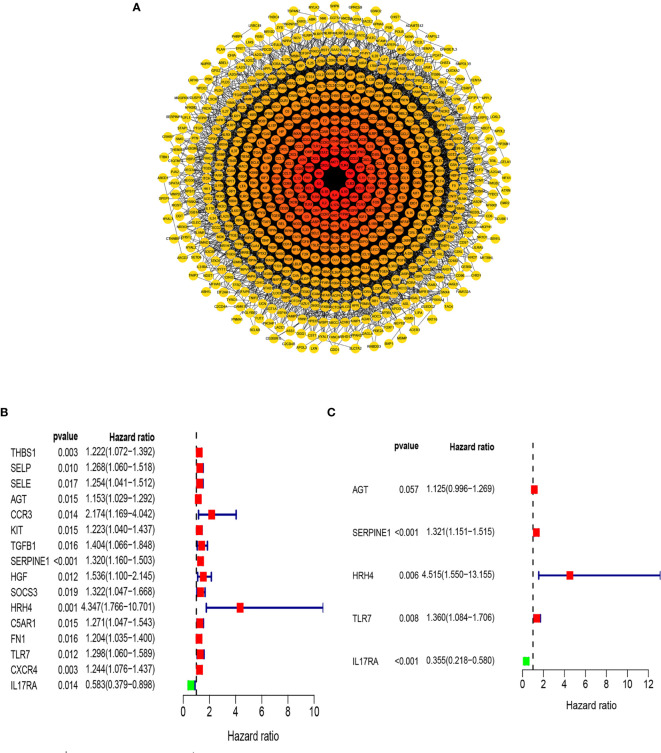
Screening of inflammation-related genes. **(A)** PPI network (interaction confidence values > 0.4). **(B)** Univariate Cox regression analysis (*P* < 0.05). **(C)** From the genes related to the prognosis of gastric cancer, the genes that are independently related to the patient’s prognosis were screened out and used to construct a multi-factor prognostic model of inflammation.

**Figure 2:**
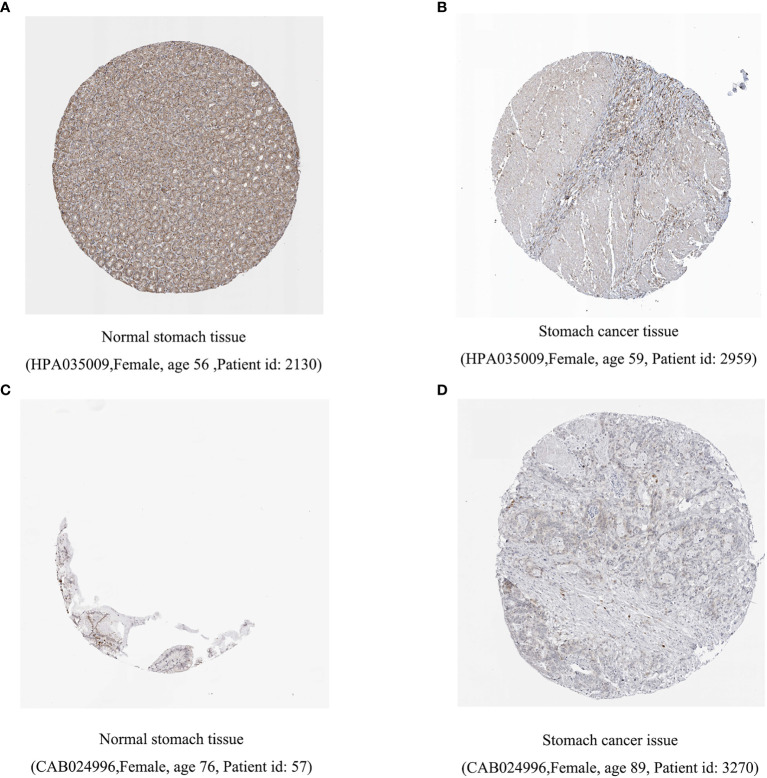
Difference in protein expression of inflammation-related genes. **(A, B)** HRH4 expression was down regulated and **(C, D)** IL-17RA expression was upregulated among gastric cancer samples in immunohistochemical images.

Previous studies have shown that inflammation and immunity play important roles in the tumor microenvironment. To explore the relationship between inflammation, immunity, and gastric cancer, we downloaded gene ontology (GO) datasets from the Gene Set Enrichment Analysis (GSEA) website to extract genes related to the inflammatory response. Next, we used the STRING PPI (http://stringdb.org/cgi/input.pl) network database and Cytoscape 3.8.2 to construct a protein interaction diagram (**Figure 1A**) of inflammatory response-related genes. The more the number of adjacent nodes in a protein, the redder the color will be, the position is closer to the center, and the protein is more important. Based on this principle, we selected the first 201 core genes that are associated with the largest number of adjacent nodes. We then downloaded the clinical information of gene expression in immune cells and immune cell infiltration profiles in gastric cancer from the TCGA database, to extract the expression of inflammatory genes in this context. Differentially expressed genes were identified by differential analysis, and Cox univariate analysis of these genes was used to screen out the inflammatory response genes most related to prognosis (**Figure 1B**, *P* < 0.02). Using this method, we identified 16 genes that were significantly related to prognosis, 15 of which were high-risk genes: *THBS1, SELP, SELE, AGT, CCR3, KIT, TGFB1, SERPINE1, HGF, SOCS3, HRH4, C5AR1, FN1, TLR7*, and *CXCR4*, whereas one, *IL17RA*, is a low-risk gene. Using COX multivariate analysis, from the aforementioned genes, we identified five genes independently affecting prognosis: *AGT, SERPINE1, HRH4, TLR7*, and *IL17RA* (**Figure 1C**). In addition, immunohistochemical images from HPA indicated low levels of HRH4 protein (**Figures 2A, B**) and high levels of IL-17RA protein (**Figures 2C, D**) in gastric cancer tissues.

### Establishment of the Prognostic Model

**Figure 3:**
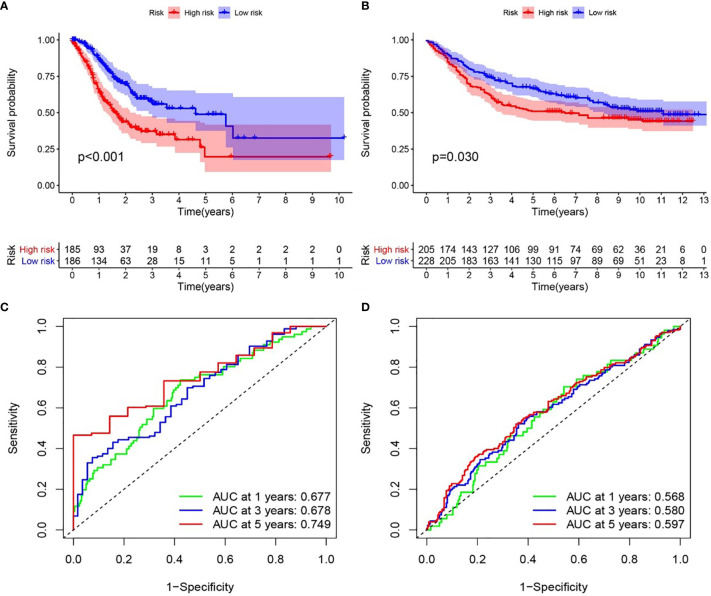
Relationship between the inflammation risk score model and patient prognosis. **(A, B)** Kaplan–Meier survival curves for patients with gastric cancer in TCGA and GEO databases stratified by high- and low-risk scores. We used the log-rank test to compare the median survival time of patients in the high- and low-risk groups (*P* < 0.001 and *P* = 0.030, respectively). **(C, D)** Analysis of the prognostic accuracy of the model through the receiver operating characteristic curve. In TCGA cohort, the areas under the 1-, 3-, and 5-year survival curves gradually increased, whereas in the GEO cohort, no significant change was observed.

**Figure 4:**
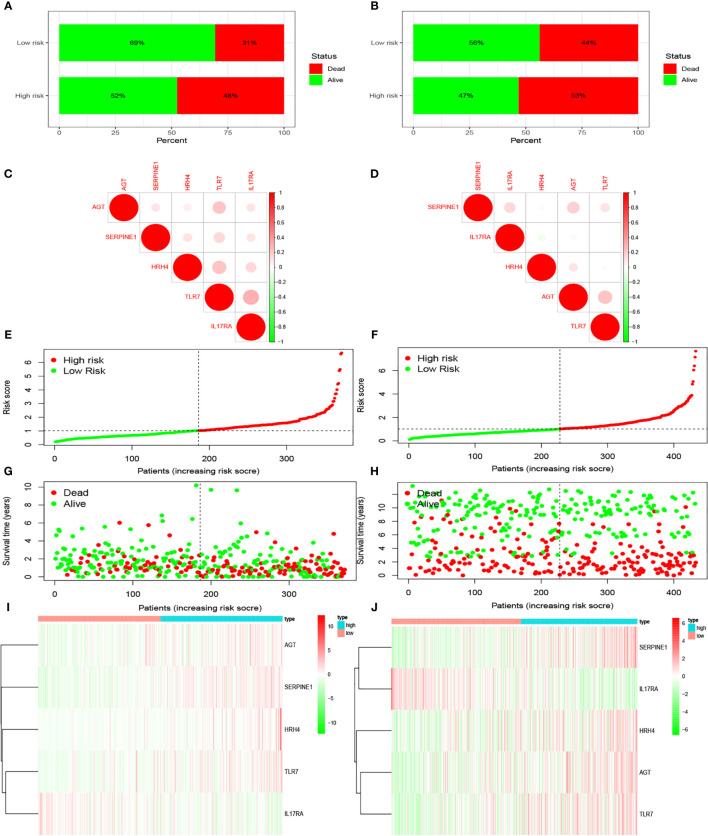
Prediction of patient risk and the expression levels of the genes included in the model in the two risk groups in TCGA and GEO databases. The patient data used were obtained from TCGA and GEO databases. **(A, B)** Patient survival. **(C, D)** Correlations between the risk model genes. Positive correlation is represented by red, and negative correlation is represented by green. **(E, F)** Patient risk scores. **(G, H)** Patient survival rates in the two risk groups. **(I, J)** Heat maps of gene expression levels in the risk models of the two risk groups.

We examined the relationship between the prognostic models and patient survival. To obtain the risk score of each patient, we multiplied the coefficients of five genes (*AGT, SERPINE1, HRH4, TLR7*, and *IL17RA*) from the GEO and TCGA databases by the corresponding expression. The gene coefficients of *SERPINE1, HRH4, AGT, TLR7*, and *IL17RA* were 0.2782, 1.5074, 0.1174, 0.3074, and -1.0345, respectively. The patients in TCGA and GEO databases were divided into two groups according to risk (high and low), and the grouping criterion was the median of the Riskscore. The subsequent survival analysis revealed that the survival status between the two groups is significantly different (**Figures 3A, B**, *P* < 0.05). The accuracy of the model for assessing survival conditions was verified using an receiver operating characteristic (ROC) curve, and the areas under the curves for 1-, 3-, and 5-year survival gradually increased in the TCGA database (**Figure 3C**). In the GEO database, the area under the ROC curve was >0.05, and the difference was not significant (**Figure 3D**), indicating that the predictive value of this model for prognosis requires improvement. A risk histogram was used to display the patient survival status in the two databases (**Figures 4A, B**). The low-risk group has a high survival rate. High- and low-risk patients can be distinguished using this model. Additionally, gene interactions in the model were analyzed (**Figures 4C, D**). By drawing the risk curve, we explored the relationship between the survival status and the risk of patients. **Figures 3E, F** show the risk value of patients in the two groups in the TCGA and GEO databases (**Figures 4E, F**). Patients in the low-risk group had a longer survival and a lower risk of death than those in the high-risk group (**Figures 4G, H**). Finally, we used a heat map to show the expression of the genes of the model in the two risk groups (**Figures 4I, J**).

### Effects of Different Clinical Traits and Riskscore on Gastric Cancer Prognosis

**Figure 5:**
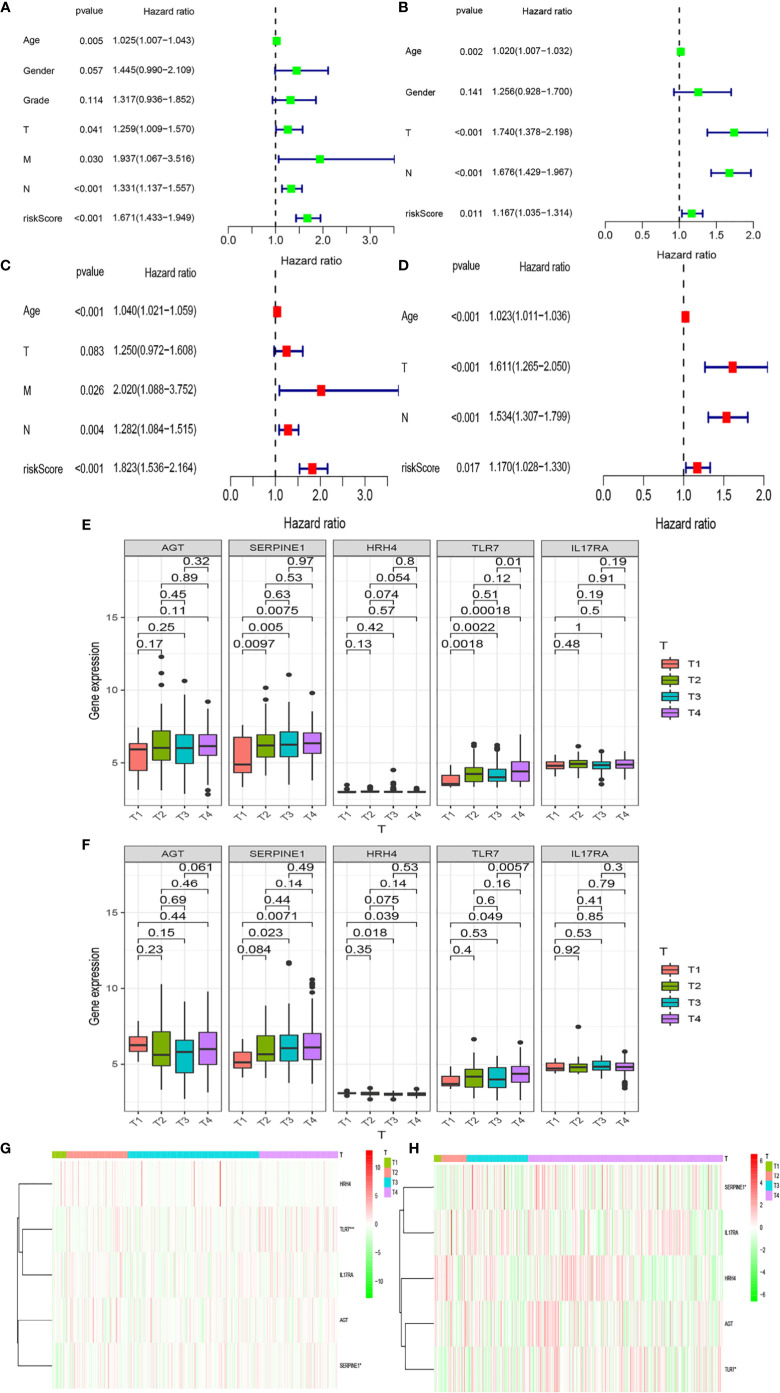
Relationships between risk models and clinical factors. Patient data were obtained from the TCGA and GEO databases. **(A, B)** Single-factor prognostic analysis including grade, gender, age, tumor– node–metastasis (TNM) stage, and the risk scores of patients. **(C, D)** Multi-factor prognostic analysis included the TNM stage, age, and risk scores. **(E, F)** The expression levels of various genes in the inflammatory model at different T stages and **(G, H)** the corresponding heatmaps.

We analyzed whether Riskscore and other clinical traits were independent prognostic factors. We explored the effects of Riskscore and different clinical traits [age, gender, and tumor–node–metastasis (TNM)] on the prognosis of patients in the two databases. Firstly, Cox univariate analysis was used to analyze the effects of clinical traits and Riskscore on the survival of patients in TCGA and GEO databases (**Figures 5A, B**). High-risk factors other than gender, such as age, T, N, and Riskscore, can influence prognosis. We also found that Riskscore influences patient prognosis: according to the results of multivariate analysis, Riskscore, age, and N are independent prognostic factors (**Figures 5C, D**). The expression of inflammatory response genes between different T stages is shown in **Figures 4E, F** (**Figures 5E, F**), and the expression of inflammatory response-related genes used to construct the prognostic model in TCGA and GEO databases at different T stages is shown in **Figures 4G, H** and **Figures 5G, H**. The differential expression of *TLR7* and *SERPINE1* at different T stages in TCGA and GEO databases was the most significant (*P* < 0.05).

### Pathway Enrichment Analysis

**Figure 6:**
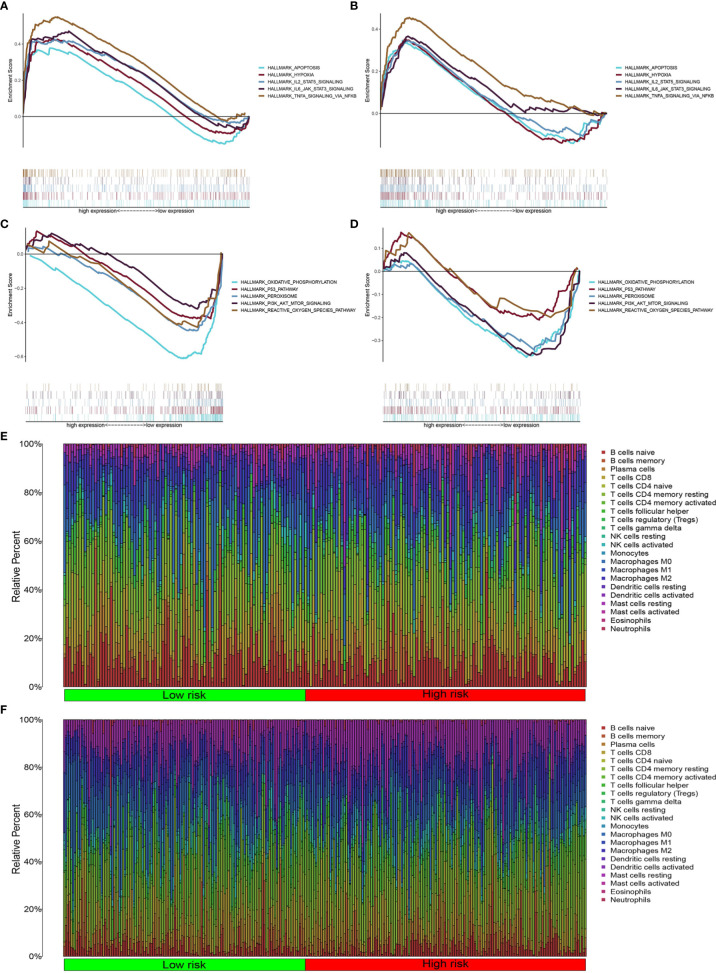
Inflammatory pathway enrichment analysis and infiltration of inflammation-related immune cells. **(A–D)** Enriched gene sets according to high- and low-risk scores in TCGA and GEO databases. Each line represents a specific gene set, with upregulated genes on the left side approaching the origin of the coordinates and downregulated genes on the right side of the x axis. Gene sets (nominal (NOM) *P* < 0.05 and false discovery rate (FDR) *q* < 0.06). A selection of leading enriched gene sets is shown. **(E, F)** Heatmap showing immune cell infiltration between high-and low-risk groups in the two databases.

We found that the high-risk groups in the two databases displayed enrichment of immune, hypoxia, and apoptotic pathways (**Figures 6A, B**), including IL-2STA T5 signaling, TNFA signaling, and IL-6-STA T3 signaling. Meanwhile, the low-risk groups were enriched with PI3K-Akt-mTOR signaling, oxidative phosphorylation, reactive oxygen species, peroxisome activity, and p53 signaling (**Figures 6C, D**).

### Immune Cell Infiltration

**Figure 7:**
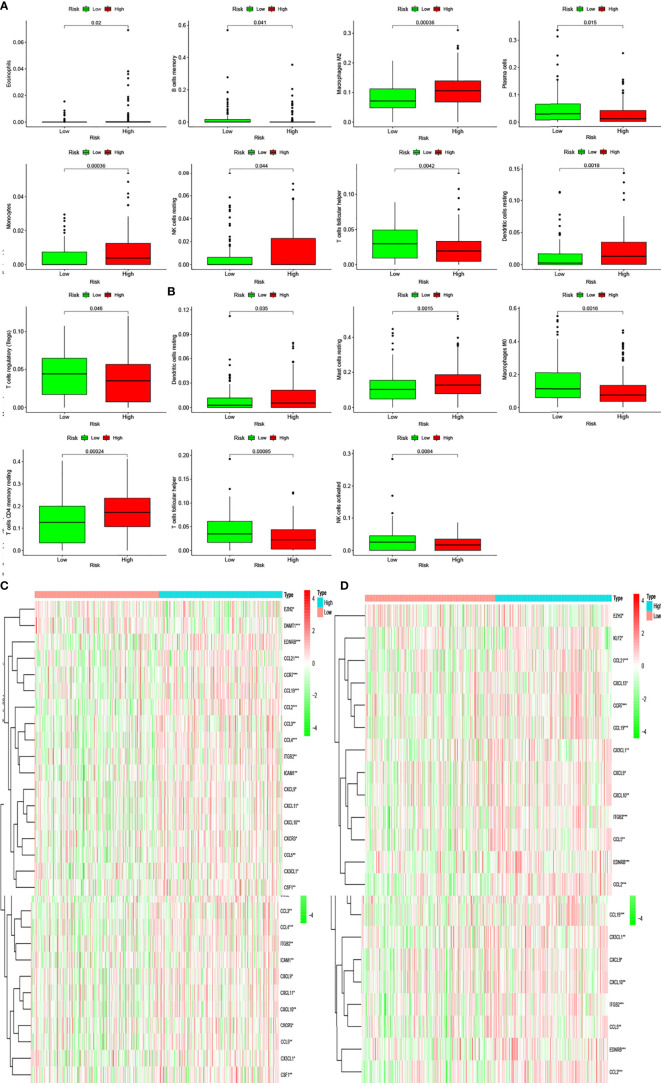
Differentially infiltrating immune cells of inflammation-associated risk groups. **(A, B)** Immune cells whose infiltration is related with inflammation risk score in the two databases (*P* < 0.05). **(C, D)** Heatmaps show the expression levels of genes regulating activated follicular helper T cells and resting dendritic cells in the two risk groups (*P < 0.05; **P < 0.01; ***P < 0.001).

Pathway enrichment analysis indicated immune factor-related pathway enrichment in both patient groups. Thus, we further clarified the infiltration of immune cells in each risk group in these two databases. **Figures 5E, F** show the infiltration of immune cells in the two risk groups in the GEO and TCGA databases, respectively (**Figures 6E, F**). In TCGA database, there were eight types of immune cells with significantly different infiltration in the two risk groups (**Figure 7A**, *P* < 0.05). In the GEO database, we found that seven types of immune cell infiltration are significantly different in the two risk groups (**Figure 7B**, *P* < 0.05).

**Figure 8:**
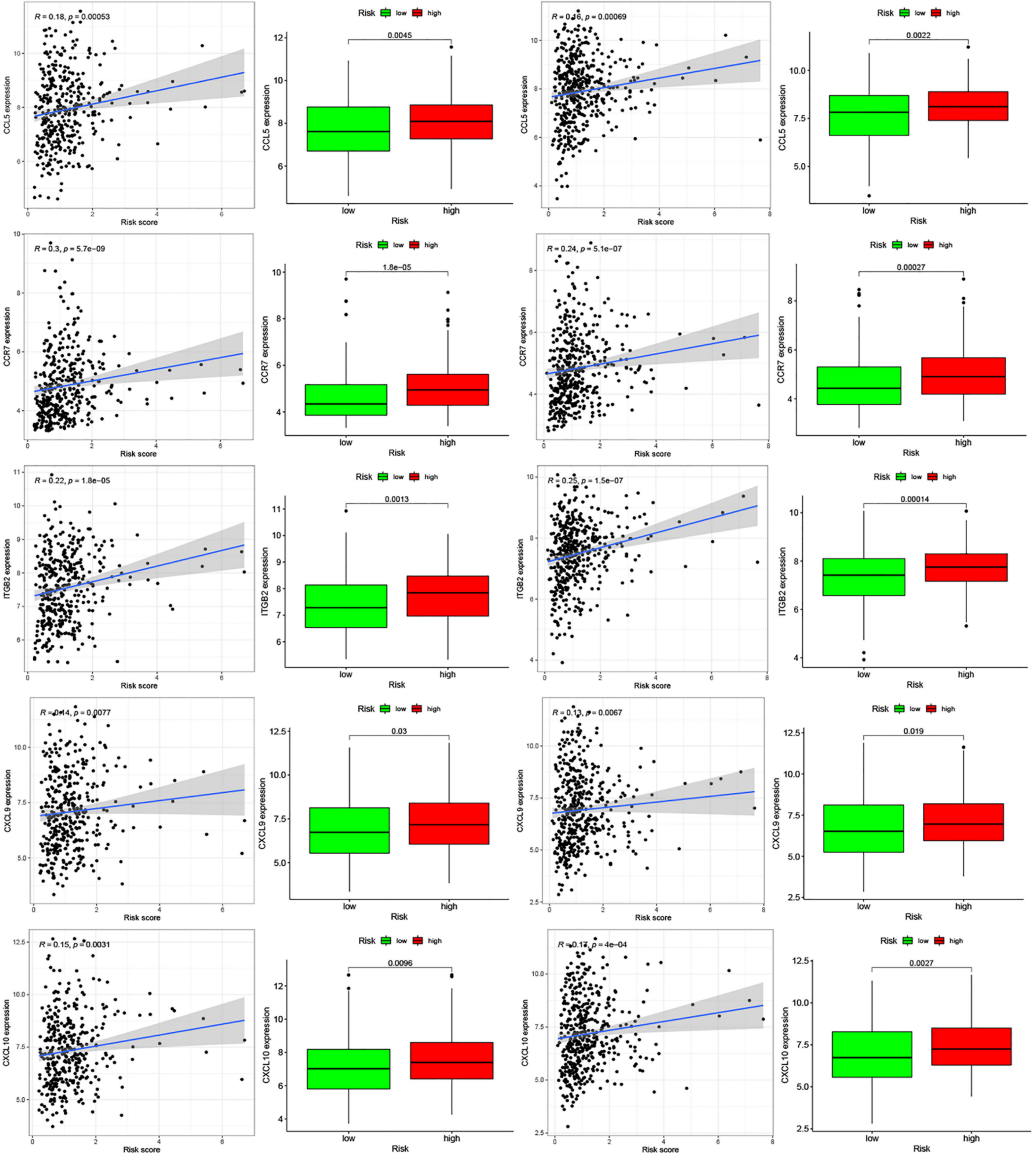
Relationships between inflammatory Riskscore and genes regulating immune cell behavior. Scatter plots show the correlations between immune cell regulation and the expression of 10 genes from the two databases. We found significant differences in the expression levels among different inflammation risk groups (*P* < 0.05). The blue line in each plot is a fitted linear model indicating the relationship between gene expression and the risk of inflammation. The box plots show the differences of gene expression between risk groups (*P* < 0.05).

**Figure 9:**
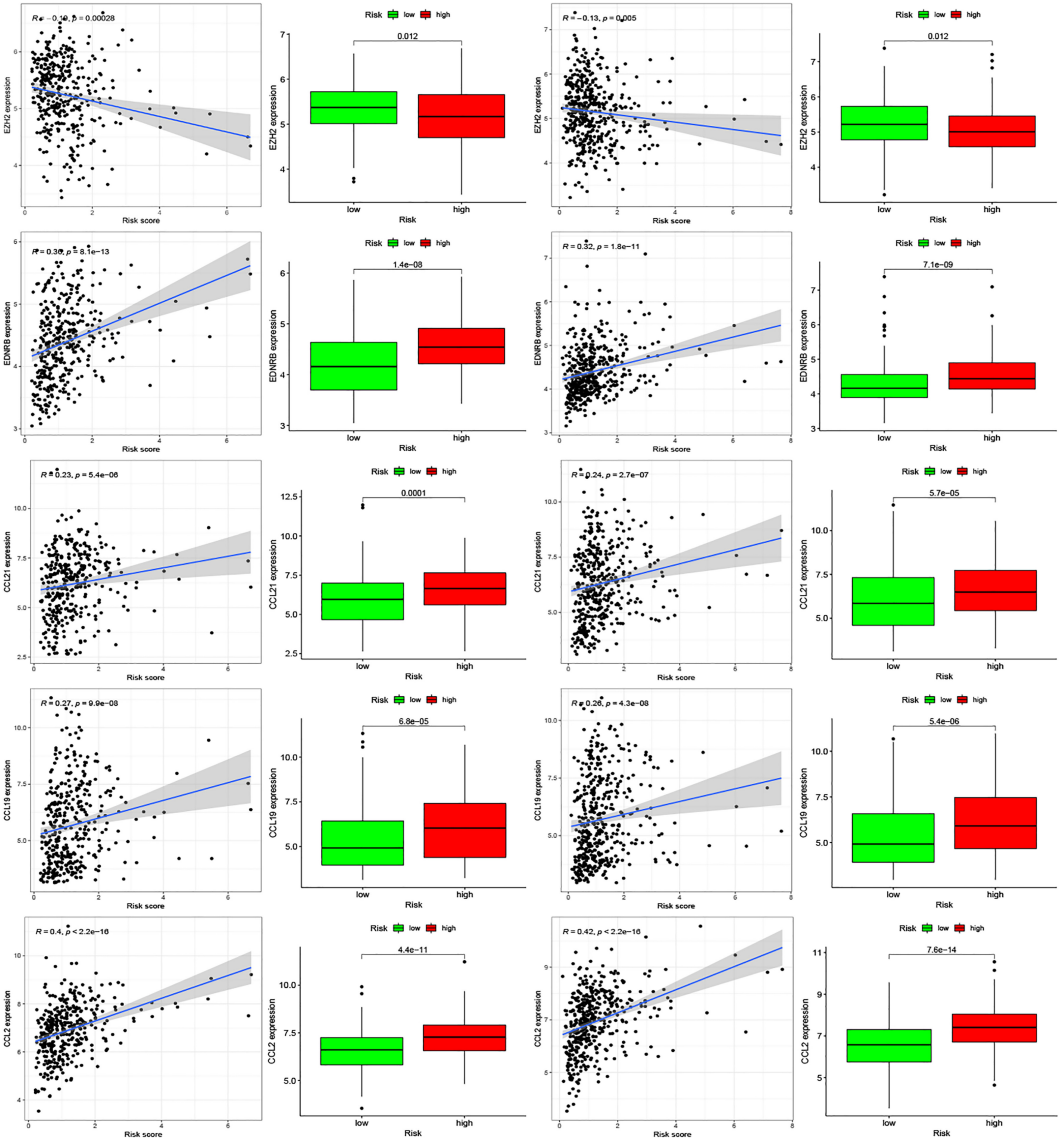
Relationships between inflammatory Riskscore and genes regulating immune cell behavior. Scatter plots show the correlations between immune cell regulation and the expression of 10 genes from the two databases. We found significant differences in the expression levels among different inflammation risk groups (*P* < 0.05). The blue line in each plot is a fitted linear model indicating the relationship between gene expression and the risk of inflammation. The box plots show the differences of gene expression between risk groups (*P* < 0.05).

Moreover, we found differences in the infiltration of follicular helper T cells and resting dendritic cells in each group in the two databases. We downloaded immune-related genes from the Tracking Tumor Immunophenotype website, and the genes regulating T cell follicular helper and dendritic cell resting were selected. The heat map shows the expression of these genes in the two risk groups of the two databases (**Figures 7C, D**). We found that the expression of 10 genes, (*EZH2, EDNRB, CCL21, CCL19, CCL2, CCL5, CCR7, ITGB2, CXCL9*, and *CXCL*) was significantly different between high- and low-risk groups in the two databases (*P* < 0.05). Next, we created a correlation curve to explore the correlation between Riskscore and the expression of these genes and found that all the 10 genes were differentially expressed in the two risk groups of TCGA and GEO databases (**Figures 8**, **9**). *EZH2* was negatively correlated with Riskscore, whereas the other genes were positively correlated. Therefore, we constructed a prognostic model of inflammation-related genes in gastric cancer that could improve the gastric cancer prognosis prediction. Moreover, we found a relationship between the prognostic model and immune cell infiltration.

## Discussion

Inflammation and stomach cancer are closely related. Inflammation plays an important role in malignant transformation (12), in which inflammation-related genes are the core of inflammation, as they act on the corresponding channel or regulating immune cells. In normal conditions, NF-κB signaling affects the regulation of inflammation and immune response. Some studies have found that the overactivation of the NF-κB pathway is a hallmark of inflammation-related cancers (13).

*Helicobacter* pylori infection is one of the main causes of chronic inflammation of the stomach, leading to the overexpression of Aurora kinase A (*AURKA*), which activates NF-κB in the gastric mucosa (14). Inflammatory factors produced by infiltrating immune cells are sustained, thereby accelerating the initiation of tumor formation (15). In this study, we screened genes related to inflammation and identified core genes to further verify the prognostic value of these genes. Except that *SELE* and *KIT* are not being studied in depth right now, we found several genes that were related to the prognosis and survival of gastric cancer patients. Among them, *AGT, SERPINE1, HRH4, TLR7*, and *IL17RA* can independently affect prognosis. Previous studies have shown that increased *SERPINE1* expression can prevent apoptosis (16). In line with this, as expression of *SERPINE1* in gastric cancer tissues is significantly increased, it is a likely contributing factor to the poor prognosis of patients with stomach cancer (17). In our study, univariate and multivariate Cox analyses showed that *SERPINE1* was a high-risk factor, which may be due to its high expression in gastric cancer patients. Previous studies have found that *TLR7* expression is reduced in stomach cancer. Increased *TLR7* expression promotes the production of proinflammatory cytokines and inhibits the growth of gastric cancer cells (18). We speculate that *TLR7* may be a high-risk factor due to its low expression in gastric cancer. Some studies show that deletion of HRH4 gene is present in gastric cancer cases and is closely correlated with attenuated gene expression. Down-regulation of HRH4 in gastric carcinomas plays a role in histamine-mediated growth control of gastric cancer cells (19). *IL17A* exerts its tumor-promoting activity through its type A receptor (IL-17RA), which is expressed in various cell types in the tumor microenvironment, including hematopoietic cells, fibroblasts, and epithelial cells (20). In addition, immunohistochemical images from HPA indicated high levels of IL-17RA protein and low levels of HRH4 protein in gastric cancer tissues. These inflammation-related genes are closely related to tumors to a large extent, which is conducive to the establishment of a subsequent inflammation model.

With an increased understanding of inflammation, scientists have recently reported on the relationship between inflammation and tumors (12, 21). In this study, we used the product sum of the expression levels and the coefficients of *AGT, SERPINE1, HRH4, TLR7*, and *IL17RA* as the risk score to evaluate patient prognosis in the TCGA and GEO databases. Patient prognosis between the two Riskscore groups was significantly different, and the patient survival in the low Riskscore group was significantly prolonged. In addition, we analyzed the effects of our Riskscore, age, sex, and T, M, and N staging on patient survival and prognosis. Riskscore has a corresponding impact on patient prognosis and can predict the prognosis of patients independent of other clinical traits.

Through pathway enrichment analysis, we found that the pathways related to apoptosis, hypoxia, and immunity were most enriched. There is often a hypoxic environment and apoptosis in tumor tissues, producing a large number of inflammatory factors. These factors can chemoattract macrophages and induce their polarization. Polarized macrophages can further produce inflammatory factors (22). Therefore, there is a close relationship between inflammation and the immune response. Several studies have previously found that inflammatory mediators can promote tumor progression and metastasis (23) and that the innate and adaptive immune systems can protect the host from tumor invasion through immune-monitoring mechanisms (24). Moreover, the increased sensitivity of immunodeficient mice to carcinogenesis and spontaneous tumors suggests that both innate and adaptive immunity can control tumor development (24–26).

In addition, we identified eight types of immune cells from TCGA database with differential infiltrations between the two patient groups. From the GEO database, in the two risk groups, the infiltration of seven immune cells was significantly different. Additionally, the infiltration of follicular helper T cells and resting dendritic cells in the two groups of the two databases was different. We then screened the genes that regulate these two cells and found that the expression of *EZH2, EDNRB, CCL21, CCL19, CCL2, CCL5, CCR7, ITGB2, CXCL9*, and *CXCL10* were different between the two risk groups. Our study also found that the expression of these genes was significantly associated with inflammatory risk scores. Six of these ten genes are chemokines, which can chemoattract immune cells and effect the development of tumors. *CCL2* and *CCL5* are closely related to prostate tumor metastasis and tumor resistance (27, 28). *EZH2* has the same characteristics as oncogenes; its overexpression *in vitro* is closely related to cell proliferation, colony formation, and benign cell invasion (29–31), and it induces xenograft tumor growth *in vivo* (32). Similarly, downregulation of *EZH2* expression in cancer cells can lead to growth arrest (31, 32), reduced tumor growth (33), and reduced metastasis (34). According to Kattan nomogram calculations, the CpG island hypermethylation of *EDNRB* is negatively correlated with the probability of survival without prostate-specific antigen (35). Meanwhile, a higher expression of *CCL21* in stomach cancer tissues is closely related to lymph node metastasis, high incidence of tumor metastasis, and depth of gastric wall invasion (36). The proliferation, migration, and invasion of stomach cancer cells are inhibited by *CCL19 via* the CCL19/CCR7/AIM2 pathway (37). Compared to healthy volunteers, the serum level of *CCL5* of gastric cancer patients was also increased, and increased exogenous *CCL5* level enhanced the migration ability of AGS cells across the transwell membrane (38). A meta-analysis study suggested that high *CCR7* expression may lead to poor survival and prognosis in patients with gastric cancer (39). Clinical studies on gastric cancer samples showed that *CXCL9* was positively correlated with better patients prognosis (40). Studies have also found that the overexpression of *CCL5* and *CXCL9* in solid tumors is associated with CD8+ T cell infiltration. In tumor tissues, T cell infiltration requires CCL5 derived from tumor cells and is amplified by CXCL9 secreted by myeloid cells induced by interferon-g (41). The invasion and migration of gastric cancer cells can be promoted by MMP-2 and MMP-9, which are upregulated by the CXCL10/CXCR3 axis (42). At present, the relationship between *ITGB2* and gastric cancer has not been thoroughly studied.

However, this study only included bioinformatics analysis. It only proves that the Riskscore model we established is related to the prognosis of gastric cancer, and the specific mechanism of the influence of inflammation-related genes on the prognosis has not been explored; thus, prospective studies, like some basic research and clinical studies should be carried out to determine the causal relationship between this model and the prognosis of gastric cancer and explore the specific mechanisms of the interaction between the genes we have identified and gastric cancer.

In conclusion, we identified inflammation-related genes related to the prognosis of gastric cancer. Our findings provide new insights on the relationship between inflammation and immunity in gastric cancer and contribute to the development of immunotherapy for the treatment of gastric cancer patients.

## Data Availability Statement

Publicly available datasets were analyzed in this study. This data can be found here: The Cancer Genome Atlas (TCGA) and GEO, GSE84437.

## Author Contributions

The data analysis and original writing of the draft were conducted by WZ and ML. SW and NZ came up with the design and critical revision of the manuscript. The original writing of the draft and its editing were by MZ, YW, and YZ. All authors contributed to the article and approved the submitted version.

## Funding

This work was supported by Finance Department of Jilin (2018SCZWSZX-039) and Finance Department of Jilin (JLSWSRCZX2020-083) and China Postdoctoral Science Foundation (2020M670034ZX).

## Conflict of Interest

The authors declare that the research was conducted in the absence of any commercial or financial relationships that could be construed as a potential conflict of interest.
